# Multimodality Imaging in Infective Endocarditis: A Clinical Approach to Diagnosis

**DOI:** 10.3390/medicina61122241

**Published:** 2025-12-18

**Authors:** Leonardo Brugiatelli, Francesca Patani, Carla Lofiego, Martina Benedetti, Irene Capodaglio, Pongetti Giulia, Francioni Matteo, Paolini Enrico, Nazziconi Marco, Kevin Maurizi, Furlani Giulia, Massari Arianna, Luciani Simone, Anselmi Benedetta, Gatti Chiara, Schicchi Nicolò, Fogante Marco, Tarsi Giovanni, Dello Russo Antonio, Di Eusanio Marco, Marini Marco, Fabio Vagnarelli

**Affiliations:** 1 “G.M. Lancisi” Cardiovascular Center, 60126 Ancona, Italy; leonardo.brugiatelli@sanita.marche.it (L.B.); francesca.patani@ospedaliriuniti.marche.it (F.P.); carla.lofiego@ospedaliriuniti.marche.it (C.L.); matteo.francioni@ospedaliriuniti.marche.it (F.M.); marco.marini@ospedaliriuniti.marche.it (M.M.); 2 San Salvatore Hospital, 61121 Pesaro, Italy; giovanni.tarsi@sanita.marche.it; 3 Cardiovascular Radiological Diagnostics, Department of Radiological Sciences, University Hospital of Marche, 60126 Ancona, Italy; nicolo.schicchi@ospedaliriuniti.marche.it (S.N.); marco.fogante@ospedaliriuniti.marche.it (F.M.); 4 Department of Biomedical Sciences, Faculty of Medicine and Surgery, Marche Polytechnic University, 60126 Ancona, Italy

**Keywords:** infective endocarditis, multimodality imaging, echocardiography, endocarditis team, cardiac CT scan, nuclear imaging

## Abstract

Infective endocarditis (IE) is a life-threatening condition with a rising incidence, demanding rapid and precise diagnosis. While echocardiography remains the cornerstone of initial evaluation, its limitations in complex cases—such as those involving prosthetic valves or cardiac devices—are well-known. This review synthesizes current evidence and guidelines to outline a practical, multimodality imaging approach for IE. We emphasize that integrating advanced techniques like cardiac computed tomography (CT) and [18F]-fluorodeoxyglucose positron emission tomography/computed tomography (FDG PET/CT) early in the diagnostic pathway, particularly in high-risk scenarios, significantly enhances diagnostic certainty, guides therapeutic decisions, and improves patient outcomes. A tailored imaging strategy, driven by clinical presentation and integrated within a multidisciplinary endocarditis team, is paramount for modern IE management.

## 1. Introduction

Infective endocarditis is a challenging disease for clinicians, with an incidence estimated at 3 to 15 cases per 100.000 persons/year in industrialized countries. Furthermore, the incidence has been rising lately. Higher rates are reported among older adults, especially those with cardiac risk factors [1,2,3], the most important being intracardiac devices, prosthetic heart valves, congenital heart diseases, prior infective endocarditis, and degenerative valvular diseases [1,4,5,6,7].

Particularly, the increased use of intracardiac devices and invasive procedures has led to a rise in healthcare-associated endocarditis, which now accounts for 33% of cases in the US [3,8] and a similar rate (26.7%) in a French population-based study [9].

Non-cardiac risk factors include hemodialysis, diabetes, immunosuppression, poor dentition, and intravenous drug use, with the latter being more frequent among younger adults [5,10].

*Staphylococcus aureus* (*S. aureus*), a Gram-positive bacterium, is the most common causative organism isolated nowadays, followed by streptococci and enterococci (with Gram-positive bacteria accounting for 80–90% of cases) [3,4,6,7,10]. Considering the aggressive behavior of *S. aureus*, a prompt diagnosis and treatment are crucial for the survival and long-term prognosis of the patient. This diagnostic imperative, however, is often complicated by nonspecific presentations and the limitations of initial imaging. Although echocardiography is the indispensable first-line tool, its sensitivity can be compromised by prosthetic material, pre-existing valve disease, and poor acoustic windows. Consequently, a significant number of cases, especially involving prosthetic valves or cardiac devices, fall into a “possible” IE category based on traditional criteria, delaying definitive therapy.

The integration of multimodality imaging—including cardiac computed tomography (CT), magnetic resonance imaging (MRI), and nuclear techniques such as [18F]-FDG PET/CT—has emerged as a paradigm shift, directly addressing these diagnostic challenges [4,8,11]. Recent guideline updates, notably from the European Society of Cardiology in 2023, have formally incorporated these advanced modalities into diagnostic criteria, reflecting their proven impact on reclassifying cases and detecting complications [8,11,12].

While previous reviews have covered technical aspects of multimodality imaging in infective endocarditis, this article focuses on practical clinical application. We go beyond listing imaging options and outline a stepwise diagnostic algorithm tailored to three main presentations: native valve, prosthetic valve, and cardiac device-related endocarditis. Importantly, this review integrates the updated 2023 ESC and ISCVID guidelines, highlighting the critical role of FDG-PET/CT and cardiac CT, and provides an actionable framework for bedside decision-making by endocarditis teams.

## 2. Materials and Methods

This comprehensive narrative review is based on a structured literature search. A systematic search of PubMed/MEDLINE, Embase, and Scopus databases was performed on 10 September 2025, for articles published between 1 January 1980 and 31 August 2025.

The search strategy was designed to be comprehensive and combined MeSH terms, EMTREE terms (for Embase), and relevant keywords based on the core concepts of “infective endocarditis” and “multimodality imaging.” The detailed, reproducible search strategy for PubMed/MEDLINE is provided in Supplementary Table S1.

Inclusion Criteria were:

- Studies on human adult subjects (age ≥ 18 years).
- Articles published in English.
- Studies focusing on the diagnostic performance, clinical utility, comparative effectiveness, or integration of echocardiography, cardiac computed tomography (CT), magnetic resonance imaging (MRI), or nuclear imaging techniques in the diagnosis and management of infective endocarditis.
- No specific diagnostic performance thresholds were required for inclusion, in keeping with the broad scope of this review.
- Priority was given to clinical guidelines, randomized controlled trials, cohort studies, case–control studies, large case series (>10 patients), and societal guidelines.
- Studies about native valve endocarditis (NVE), prosthetic valve endocarditis (PVE), and/or cardiac implantable electronic device (CIED) endocarditis were all considered eligible.

Exclusion Criteria were:

- Studies conducted exclusively on pediatric populations.
- Studies not published in English.
  - Studies not primarily focused on cardiac imaging or where the relevant imaging data could not be extracted.
  - Studies focusing exclusively on non-cardiac or highly specific cardiac devices (e.g., ventricular assist devices, left atrial appendage occluders, transcatheter edge-to-edge repair systems) were excluded, as the available evidence was limited and their inclusion would have made the scope of the review too broad.

The initial search results were screened by title and abstract by two independent reviewers (L.B. and F.P.) to identify potentially eligible studies. The full text of these articles was then assessed against the inclusion and exclusion criteria. Additional relevant sources were identified through manual screening of reference lists from key articles and recent clinical guidelines.

A formal meta-analysis was deemed inappropriate due to the significant clinical and methodological heterogeneity among the identified studies (e.g., variations in patient populations, imaging protocols, and reference standards). Therefore, a narrative synthesis was conducted to provide an integrated, clinically oriented perspective.

To ensure the review reflects the most current evidence, a targeted supplementary search was conducted during the revision phase (up to October 2025) for key updates (particularly related to Artificial Intelligence, Machine Learning, and Blood Culture-Negative Endocarditis). Relevant articles identified were incorporated into the final manuscript.

Generative artificial intelligence tools were used during the preparation of this manuscript for specific, limited purposes. Grammarly (Grammarly, Inc.; San Francisco, California, USA; Software version: Grammarly 1.144.1.0) was employed for proofreading and grammar checking. DeepSeek (DeepSeek, Beijing, China; Software version: DeepSeek-V2) was used to create and refine the summary figures and diagnostic pathways through iterative prompt engineering. All AI-generated content, including visual representations, underwent critical review, fact-checking against primary sources, and substantial editing by the authors to ensure scientific accuracy, appropriate contextualization, and compliance with academic standards. The final manuscript reflects the authors’ intellectual input and supervision throughout the development process. No AI tool replaced any part of the systematic literature search, data extraction, critical analysis, or clinical interpretation.

All clinical images presented in this review (Figure 1, Figure 2, Figure 3, Figure 4, Figure 5, Figure 6, Figure 7, Figure 8, Figure 9, Figure 10, Figure 11, Figure 12, Figure 13, Figure 14, Figure 15, Figure 16, Figure 17, Figure 18 and Figure 19) are original, de-identified cases from patients treated at our institution. Written informed consent for the publication of these de-identified images was obtained from all patients. The use of these images for publication was approved by the Institutional Review Board of the Marche Polytechnic University.

All diagrams, flowcharts, and graphical abstracts (Figure 20 and Figure 21) were created for this publication.

## 3. Diagnosis of Infective Endocarditis

**Figure 1:**
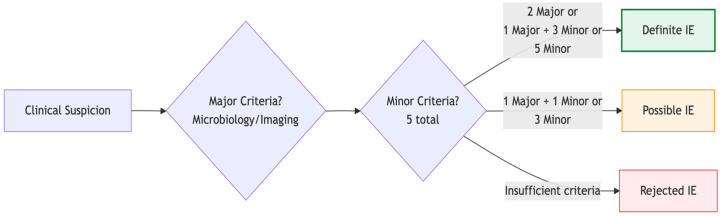
The diagnosis hinges on combinations of major criteria (microbiology/imaging) and minor clinical criteria, with classification as definite, possible, or rejected IE determining subsequent management.

The modified Duke criteria for infective endocarditis (IE) classify cases as “definite,” “possible,” or “rejected” IE (Figure 1). Classification is based on a combination of major and minor criteria. The major criteria include:

(1) **Typical** microorganisms consistent with IE from two separate blood cultures: Oral streptococci, Streptococcus Gallolyticus (formerly *S. Bovis*), HACEK group, *S. aureus*, *E. faecalis*. Specifically, the sample might be acquired through:
  - two separate blood cultures (drawn 30 min apart or 12 h apart) or
  - all of 3 or a majority of ≥4 separate cultures of blood (with first and last samples drawn ≥1 h apart).

Or a single positive blood culture for Coxiella *Burnetii* or antiphase I IgG antibody titer > 1:800.

(2) **Evidence of infective endocardial involvement demonstrated by imaging findings** such as valvular, perivalvular/periprosthetic and foreign material anatomic and metabolic lesions characteristic of IE. These lesions can be detected by any of the following imaging techniques: echocardiography (TTE and TEE), cardiac CT, [18F]-FDG-PET/CT(A) and WBC SPECT/CT.

In challenging cases, the 2023 updated Duke-ISCVID (International Society for CardioVascular Infectious Diseases) criteria recognized molecular diagnostics, such as polymerase chain reaction, amplicon/metagenomic sequencing, and in situ hybridization, as **major criteria**, especially for culture-negative cases [2].

The minor **criteria for infective endocarditis are**:

- Predisposing heart condition (such as having a prosthetic heart valve or previous heart infection) or injection drug use
- Fever above > 38 °C (100.4 °F)
- Vascular phenomena (such as major arterial emboli—blockages in large arteries, septic pulmonary infarcts—infections causing dead lung tissue, mycotic aneurysm—a blood vessel swelling caused by infection, intracranial hemorrhage—bleeding in the brain, conjunctival hemorrhages—bleeding in the eye, or Janeway lesions—painless red or purple skin spots)
- Immunological phenomena (such as glomerulonephritis—a type of kidney inflammation, Osler’s nodes—tender bumps on fingers or toes, Roth’s spots—retinal hemorrhages in the eye, or a positive rheumatoid factor—a blood antibody).
- Microbiological evidence not meeting major criteria (positive blood culture or serological evidence—blood test showing immune response—infection with an organism consistent with Infective endocarditis.

The American College of Cardiology (ACC), European Society of Cardiology (ESC), and American Heart Association (AHA) recommend these criteria (Modified Duke Criteria) because they integrate clinical, microbiological, and imaging findings, providing high sensitivity and specificity for diagnosis across diverse patient populations, including those with prosthetic valves and injection drug use [2,13,14].

As an example, matrix-assisted laser desorption/ionization time-of-flight (MALDI-TOF) mass spectrometry is a rapid, culture-based technique for microbial identification that has become increasingly relevant in the diagnosis of infective endocarditis (IE), especially in cases with negative blood cultures or atypical pathogens. MALDI-TOF is recognized as a valuable adjunct for pathogen identification from positive blood cultures and excised valve tissue, facilitating early and accurate microbiological diagnosis in IE [13].

The 2023 update by the ISCVID also expanded the imaging criteria, incorporating advanced modalities such as cardiac CT and 18F-FDG PET/CT, especially for prosthetic valves and intracardiac devices [14,15,16].

Imaging plays a central role in fulfilling the primary diagnostic criteria. Transthoracic and transesophageal echocardiography remain first-line, but their sensitivity is limited in patients with prosthetic valves or intracardiac devices due to acoustic shadowing and device-related artifacts. In these populations, multimodality imaging—particularly cardiac CT and 18F-FDG PET/CT—improves sensitivity and diagnostic yield, as recognized by the European Society of Cardiology (ESC) and other societies [4,5]. These modalities can detect perivalvular complications, prosthetic dehiscence, and metabolic activity suggestive of infection, which are often missed by echocardiography alone [12,16,17,18,19].

Limitations persist: imaging specificity may be reduced by non-infectious causes of increased metabolic activity (e.g., post-surgical inflammation), and feasibility can be affected by patient comorbidities or device type. Interobserver variability and reduced specificity, especially with PET/CT, are notable concerns in prosthetic valve endocarditis [3,9]. Thus, while advanced imaging is now integral to diagnosis, interpretation requires careful clinical correlation and awareness of modality-specific pitfalls [14,19].

### Blood Culture-Negative Endocarditis

In blood culture–negative endocarditis (BCNE), the absence of microbiological confirmation places paramount importance on advanced imaging modalities to establish the diagnosis. When echocardiography (TTE/TEE) is inconclusive or clinical suspicion remains high, cardiac CT and FDG-PET/CT become critical first-line diagnostic tools. These modalities can detect vegetations, abscesses, pseudoaneurysms, and extracardiac infectious foci that may be missed by ultrasound. Reflecting their central role, such imaging findings now constitute major criteria in updated diagnostic algorithms (e.g., the 2023 Duke-ISCVID criteria) [20]. FDG-PET/CT is particularly valuable not only for identifying metabolically active infections but also for helping to differentiate them from non-infectious mimics. For instance, nonbacterial thrombotic endocarditis (NBTE or Libman-Sacks endocarditis), associated with autoimmune conditions like systemic lupus erythematosus, typically shows minimal or absent FDG uptake—a key distinguishing feature [20,21].

Importantly, the anatomical or metabolic lesions identified by these advanced imaging techniques can then guide targeted microbiological investigation. Findings suggestive of IE on CT or PET/CT should prompt further serological testing (e.g., for *Coxiella burnetii*, *Bartonella* spp.) or molecular analysis (e.g., PCR, sequencing) on excised valve tissue to identify fastidious organisms such as those from the HACEK group, *Bartonella* species, or fungi [20]. Therefore, in BCNE, advanced imaging serves a dual purpose: securing the anatomical diagnosis of endocarditis and directing the subsequent search for the causative pathogen.

## 4. Echocardiography

Transthoracic echocardiography (TTE) is the recommended first-line imaging modality for the initial evaluation of suspected infective endocarditis (IE), due to its noninvasive nature, wide availability, and ability to rapidly assess cardiac structures for vegetations, valvular dysfunction, and complications such as abscess or prosthetic valve dehiscence [8,22,23].

Echocardiographic masses that are suggestive of endocarditis are most commonly vegetations, which are defined as oscillating intracardiac masses attached to cardiac valves or other endocardial structures, typically on the low-pressure side of the valve structure (but may be located anywhere on the components of the valvular and subvalvular apparatus) or on implanted material, and without an alternative anatomic explanation [4,24]. Echocardiographic findings highly suggestive of endocarditis include:

- valvular or leaflet perforation, i.e., tissue defects causing valvular regurgitation originating from the site of perforation.
- valvular aneurysm, a saccular outpouching of a valve leaflet, protruding into the atrium or ventricle.
- perivalvular or perigraft abscess, an echolucent or echodense area adjacent to the valve annulus or prosthetic ring often with irregular borders and sometimes with evidence of cavity formation.
- pseudoaneurysm, a contrast-filled outpouching with a narrow neck communicating with the cardiac lumen, often adjacent to the valve annulus. On echocardiography, it appears as a pulsatile cavity with systolic expansion and diastolic collapse.
- intracardiac fistula, visualized as an abnormal communication between cardiac chambers or vessels, is often detected by using color Doppler.
- significant new valvular regurgitation compared with previous imaging, i.e., increase in regurgitant jet size, vena contracta width…

The sensitivity of TTE for detecting vegetation in native valve endocarditis is approximately 60–70%, whereas it is lower (about 50–58%) in prosthetic valve endocarditis due to acoustic shadowing and limited visualization of perivalvular regions. Specificity is high, typically 94–98%, making a positive TTE highly suggestive of IE [4,8,22,25].

**Figure 2:**
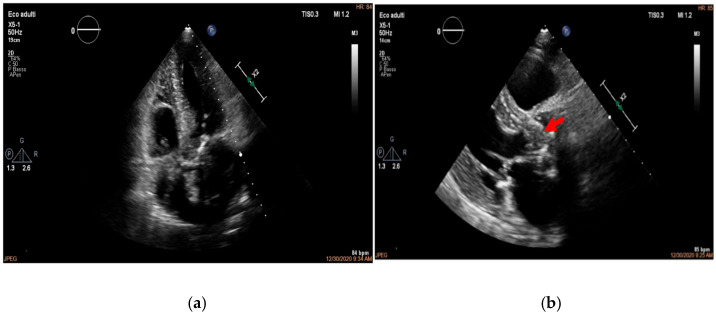
(**a**,**b**). Transthoracic echocardiogram showing aortic valve vegetation, more evident on the ventricular side of the aortic valve, as indicated by the arrow. This is an original, de-identified clinical image from our institution. Patient consent for publication was obtained.

**Figure 3:**
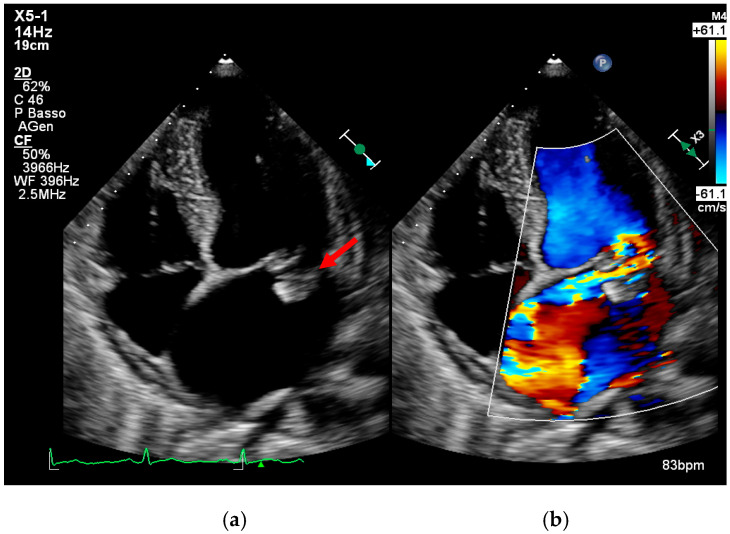
(**a**,**b**). Transthoracic echocardiogram showing mitral valve vegetation (arrow, **a**), causing perforation of the left posterior mitral valve leaflet and severe regurgitation (**b**). This is an original, de-identified clinical image from our institution. Patient consent for publication was obtained.

**Figure 4:**
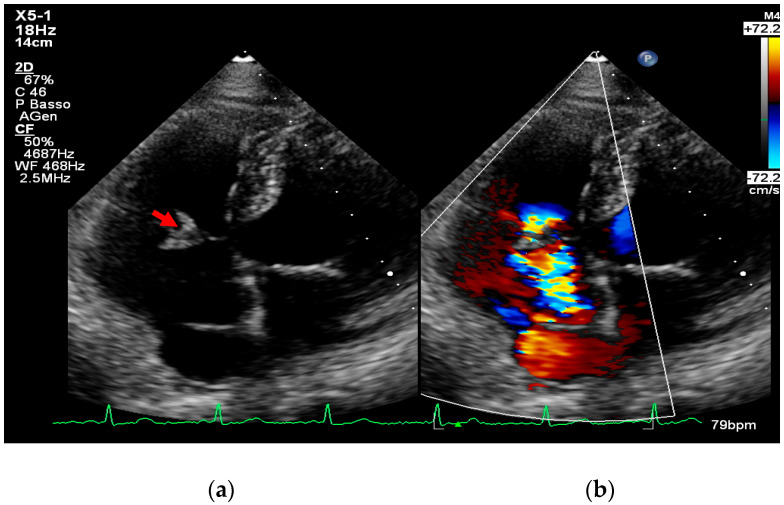
(**a**,**b**). Transthoracic echocardiogram showing tricuspid valve vegetation (arrow, **a**) and severe regurgitation (**b**) caused by infectious damage to the septal leaflet. This is an original, de-identified clinical image from our institution. Patient consent for publication was obtained.

Importantly, when TTE images are of high quality and show no abnormalities, the negative predictive value can reach 95–97%, especially in patients without prosthetic valves or high-risk features [8,25] (Figure 2, Figure 3 and Figure 4).

On **prosthetic valves**, vegetations are more challenging to detect due to acoustic shadowing and reverberation artifacts from the prosthesis. When present, vegetations tend to form around the valve ring (sewing ring or annulus) and may extend to the prosthetic leaflets, stent, or occluders. They are often **smaller** and **less mobile** than those on native valves. Additionally, prosthetic valve endocarditis **is more likely to present with paravalvular complications** such as abscesses, pseudoaneurysms, and dehiscence, which may appear as echo-lucent or echo-dense masses or abnormal cavities adjacent to the prosthesis, sometimes without visible vegetations [26]. Masses of different nature (e.g., thrombus, suture material) need to be distinguished through careful clinical correlation.

**Figure 5:**
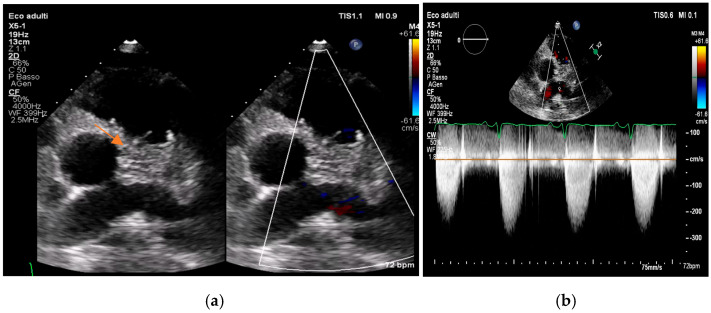
(**a**,**b**): Endocarditis of the pulmonary bio-conduit (arrow) in a patient with a history of injection drug use and previous endocarditis of the native pulmonary valve, which led to valve replacement (**a**). Anterograde gradient was elevated (peak: 34 mmHg, mean: 24 mmHg), resulting in moderate stenosis (shown on the right in **b**). This is an original, de-identified clinical image from our institution. Patient consent for publication was obtained.

Particularly, the tricuspid valve and the pulmonic valve are anterior structures; as such, they tend to be accurately defined with TTE, as in Figure 4 and Figure 5.

Limitations of TTE include reduced sensitivity in patients with obesity, chronic obstructive pulmonary disease, chest wall deformities, or prosthetic valves, and inability to reliably detect small vegetations or paravalvular complications such as abscesses [4,5,8].

False negatives may occur early in the disease, in cases of recent antibiotic treatment or if vegetations have embolized [15,22].

**Figure 6:**
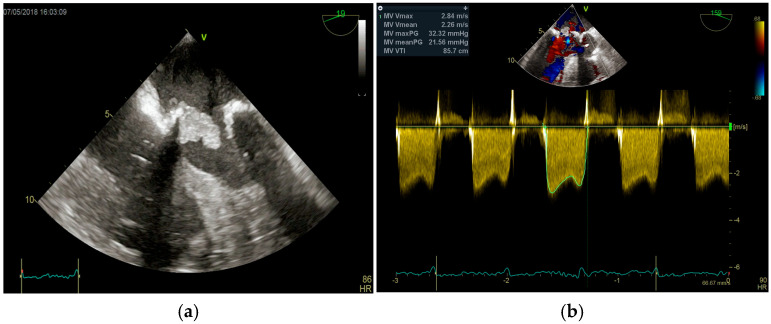
(**a**,**b**): Huge mitral valve endocarditis (**a**) determining fixed anterograde flow obstruction (mitral stenosis with Doppler tracing shown on the right). This is an original, de-identified clinical image from our institution. Patient consent for publication was obtained.

In cases of high clinical suspicion, poor image quality, or presence of prosthetic material, TEE is indicated for its superior sensitivity (up to 95%) and comparable specificity [8,22,27] (Figure 6).

**Figure 7:**
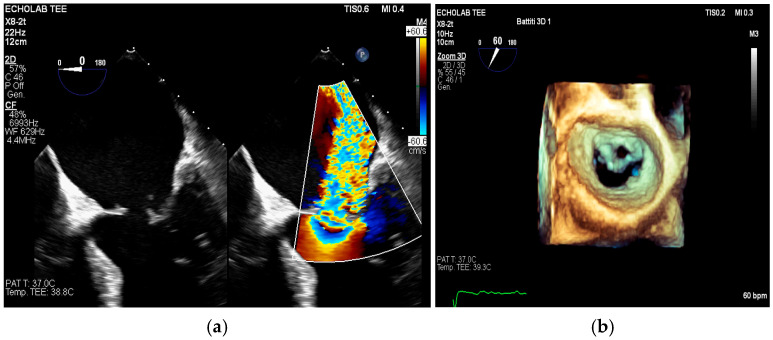
(**a**,**b**): On the left, leaflet perforation is suspected from the Color Doppler pattern observed (**a**). On the right (**b**), the 3D zoom further defines the origin of the perforation. This is an original, de-identified clinical image from our institution. Patient consent for publication was obtained.

**Figure 8:**
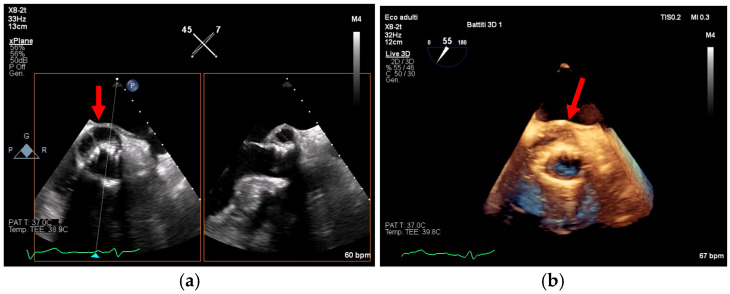
(**a**,**b**): These figures show a cavity with systolic expansion near the anterior portion of a newly implanted transfemoral aortic bioprosthesis (arrow). Three-dimensional imaging confirms the anterior position of this defect (arrow) and helps localize it accurately, defining its extension. This is an original, de-identified clinical image from our institution. Patient consent for publication was obtained.

**Figure 9:**
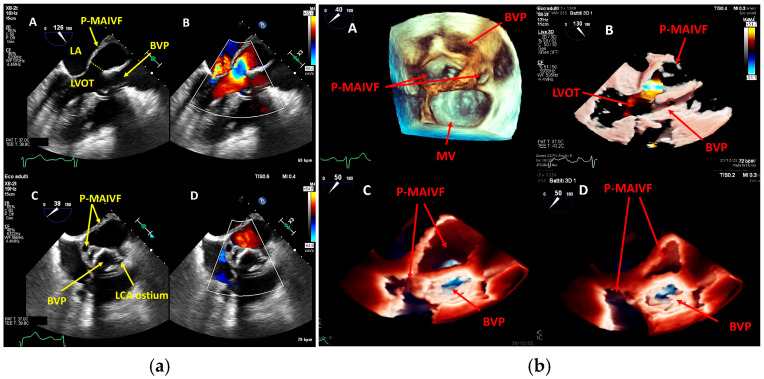
(**a**,**b**): On the left (**a**), a pseudoaneurysm of the Mitral-Aortic Intervalvular Fibrosa (P-MAIVF) is visible. The cavity demonstrates systolic expansion (A, C) and color Doppler flow (B, D) directed inward during systole. On the right (**b**), the 3D echo view is displayed (A: surgical view, B, C, D: “TrueVue Glass” view helps delineate the anatomical borders of the cavity). LCA = Left Coronary Artery. BVP = Biological Valve Prosthesis; LVOT = Left Ventricular Outflow Tract. LA = Left Atrium. This is an original, de-identified clinical image from our institution. Patient consent for publication was obtained.

TEE offers superior spatial resolution compared to transthoracic echocardiography (TTE). It is also markedly superior for identifying paravalvular complications such as abscesses, fistulae, leaflet perforation, and prosthetic valve dehiscence, which are critical for surgical decision-making and prognosis [8,24,27]. Three-dimensional echocardiography, in particular, could be used to best define lesion entity and location (Figure 7, Figure 8 and Figure 9).

Furthermore, a key indicator of perivalvular involvement is the presence of new atrioventricular (AV) block or bundle branch block. The AHA states that a new AV block has a high positive predictive value (88%) for perivalvular abscess. However, its sensitivity is limited (45%)—meaning its presence is highly suggestive, but its absence does not rule out perivalvular involvement [24]. In clinical practice, the detection of new AV blocks should prompt immediate escalation of diagnostic imaging [28].

The American College of Cardiology further emphasizes that a single negative TEE does not exclude endocarditis if clinical suspicion persists. In such cases, a repeat TEE may be warranted, along with a cardiac CT scan [3].

TEE is also valuable intraoperatively for reassessing anatomy, guiding surgical management, and monitoring new complications that may arise during the course of the disease [3].

In summary, TEE is essential for definitive diagnosis, assessment of complications, and guiding management in infective endocarditis, especially in patients with new AV block, prosthetic valves, intracardiac devices, or high clinical suspicion despite initial inconclusive studies.

**Figure 10:**
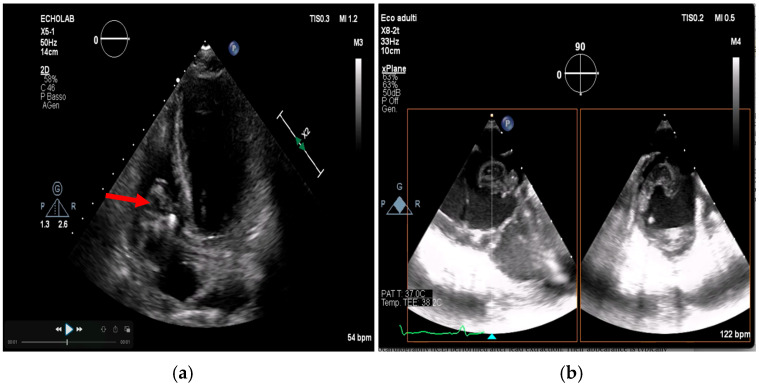
(**a**,**b**): Large CIED endocarditis detected with TTE, surrounding the sinus catheter of a cardiac resynchronization device (CRT—arrow, **a**), later confirmed by TEE (shown on the right—**b**). This is an original, de-identified clinical image from our institution. Patient consent for publication was obtained.

Finally, echocardiography is the primary imaging modality for diagnosing cardiac implantable electronic device (CIED) endocarditis. Both transthoracic echocardiography (TTE) and transesophageal echocardiography (TEE) are recommended in patients with suspected CIED-related infective endocarditis, with TEE offering superior sensitivity for detecting lead-associated vegetations, valve involvement, and complications (Figure 10) [24,29].

TEE cannot reliably distinguish infected vegetations from sterile thrombi or noninfected masses on device leads, and a negative echocardiogram does not exclude CIED endocarditis, as lead infection may be present without visible masses. Repeat echocardiography or adjunctive imaging modalities may be necessary if clinical suspicion remains high despite initial negative findings [29,30].

After CIED removal, a particular echocardiographic finding may be present: ghosts.

**Figure 11:**
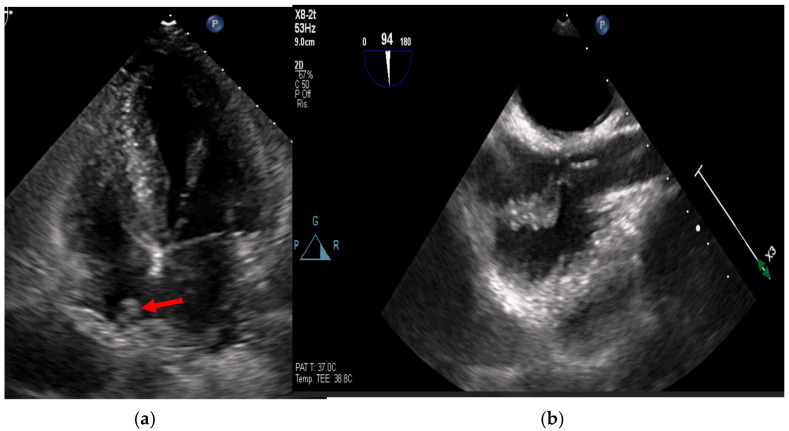
(**a**,**b**): ghost detected in this TTE on the roof of the right atrium (arrow), in a patient who underwent a recent CIED extraction (**a**). On the right, the corresponding TEE bicaval view (**b**). This is an original, de-identified clinical image from our institution. Patient consent for publication was obtained.

“Ghosts” on echocardiography are residual, tubular, mobile intracardiac masses that follow the route of the extracted lead, typically visualized in the right atrium or ventricle after transvenous lead extraction. These structures represent fibrotic tissue or organized thrombus that remains after device removal, and are not active vegetations or retained hardware [31,32,33] (Figure 11).

Their appearance is typically that of a mobile, tubular mass, sometimes extending along the previous lead tract, and they are more frequently observed in cases with longer lead dwell time, lead abrasions, or extraction for infectious indications [31].

Ghosts have prognostic implications: their detection post-extraction is independently associated with increased mid-term mortality and adverse events, including embolic complications and the need for further surgical intervention [32].

However, in the absence of ongoing infection or embolic events, ghosts are deemed benign, as reported in case reports and cohort studies [34].

If there is clinical suspicion of ongoing infection or if echocardiographic findings are equivocal, advanced imaging modalities such as FDG-PET/CT may be considered to evaluate for residual infection, particularly in cases of incomplete device removal or persistent bacteremia [8,29,35]. The optimal timing for follow-up imaging is not standardized, but a delay of 4–6 weeks post-extraction may reduce false positives due to post-procedural inflammation [29,35].

### Differential Diagnosis

**Figure 12:**
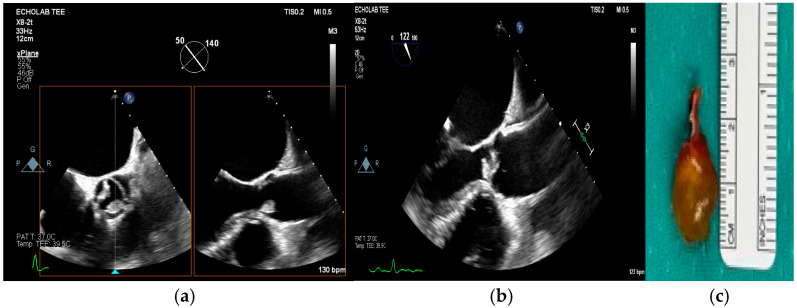
(**a**–**c**): In these figures, the image present at the edges of the aortic valve could resemble an endocarditis (**a**,**b**), but clinical features were not concordant (absence of fever, no embolic lesions, no predisposing factors). The mass was well-rounded and was later defined as a fibroelastoma following cardiac surgery and anatomical examination (**c**). This is an original, de-identified clinical image from our institution. Patient consent for publication was obtained.

Several echocardiographic pitfalls can mimic infective endocarditis, leading to false-positive interpretations. Common mimics include Lambl excrescences (filiform, mobile structures on valve leaflets), valve sclerosis or calcification, myxomatous degeneration, papillary fibroelastomas, and nonbacterial thrombotic endocarditis. Additionally, thrombus, pannus formation (especially on prosthetic valves), and caseous mitral annular calcification may resemble vegetations or abscesses. Normal anatomic variants, such as the Chiari network or redundant chordae, can also be misinterpreted as pathological findings (Figure 12) [13,17,23,36].

The ESC, AHA, and ISCVID highlight that previous scarring, severe myxomatous change, and normal structures, such as Lambl excrescences, may be indistinguishable from active endocarditis on echocardiography. High-resolution TEE imaging and repeat studies can help clarify indeterminate findings, especially when rapidly moving structures or microcavities are present. The location, attachment, mobility, and echogenicity of the lesion, as well as clinical context, are critical for differentiation.

In prosthetic valves, distinguishing between vegetation, thrombus, and pannus is particularly challenging due to imaging artifacts and overlapping features. Advanced modalities such as cardiac CT or FDG-PET/CT may be considered when echocardiographic findings are inconclusive.

## 5. Cardiac Computed Tomography

**Figure 13:**
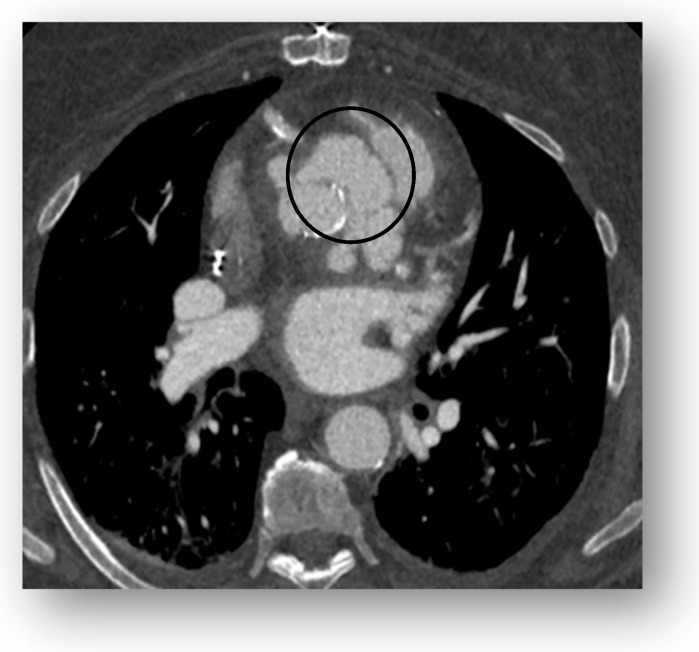
This picture shows an aortic root endocarditis complicated by a pseudoaneurysm (circle) suspected on TTE, later confirmed by CT scan. This is an original, de-identified clinical image from our institution. Patient consent for publication was obtained.

Cardiac computed tomography (CT) is recommended as an adjunct imaging modality in the evaluation of infective endocarditis (IE, especially prosthetic valve endocarditis [PVE]) when echocardiography is inconclusive or inadequate. The American College of Cardiology and American Heart Association (ACC/AHA) guidelines assign cardiac CT a class IIa recommendation for suspected paravalvular abscess in cases with non-diagnostic echocardiography. The European Society of Cardiology (ESC) guidelines give it a class I indication for detecting valvular lesions, confirming possible PVE, and identifying periprosthetic complications (Figure 13) [13].

**Figure 14:**
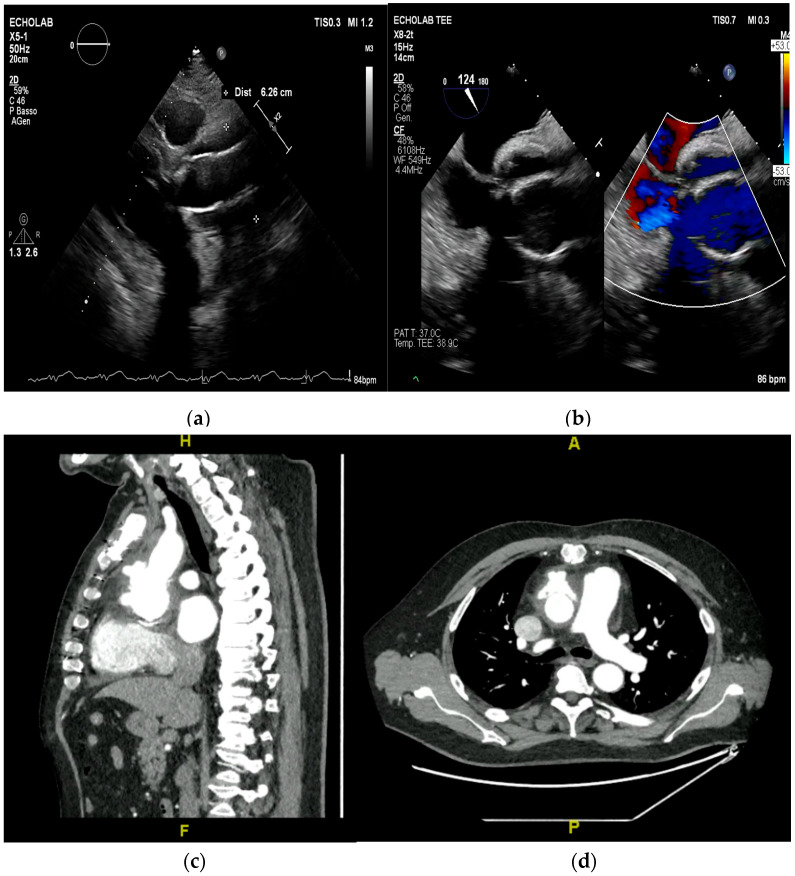
(**a**–**d**): These images show a detachment of a valved conduit that was previously implanted for severe aortic regurgitation and aortic root enlargement (**a**,**b**). In (**c**,**d**) the CT scan further defines the anatomy of the endocarditis, which is particularly useful in presurgical planning. This is an original, de-identified clinical image from our institution. Patient consent for publication was obtained.

Cardiac CT is less sensitive than transesophageal echocardiography (TEE) for detecting small, mobile vegetations. Still, it is particularly valuable for delineating the extent of infection, identifying periannular abscesses, pseudoaneurysms, and mycotic aneurysms, as well as for preoperative planning. CT is less affected by prosthetic valve artifacts (e.g., shadowing from mechanical valves or sewing rings) than ultrasound, allowing improved visualization of perivalvular structures and complications (Figure 14).

CT can also detect distant embolic phenomena and, when combined with PET imaging, provides both anatomical and metabolic information [37].

The rationale for these recommendations is that CT offers superior anatomical detail for perivalvular and extracardiac complications, which are common in PVE and may be missed by echocardiography due to acoustic shadowing or reverberation artifacts. CT is also useful for assessing coronary artery status in patients with IE who may require reoperation [26,37].

## 6. Magnetic Resonance

Magnetic resonance imaging (MRI) plays a complementary role in the evaluation of infective endocarditis (IE), particularly for the detection of extracardiac complications such as cerebral embolic events, many of which are clinically silent. The American Heart Association and Infectious Diseases Society of America note that MRI has had a significant impact on IE management in this context. However, routine indications for cardiac MRI in IE are not well established [24].

### 6.1. The Primary Role: Detection of Embolic Complications

**Figure 15:**
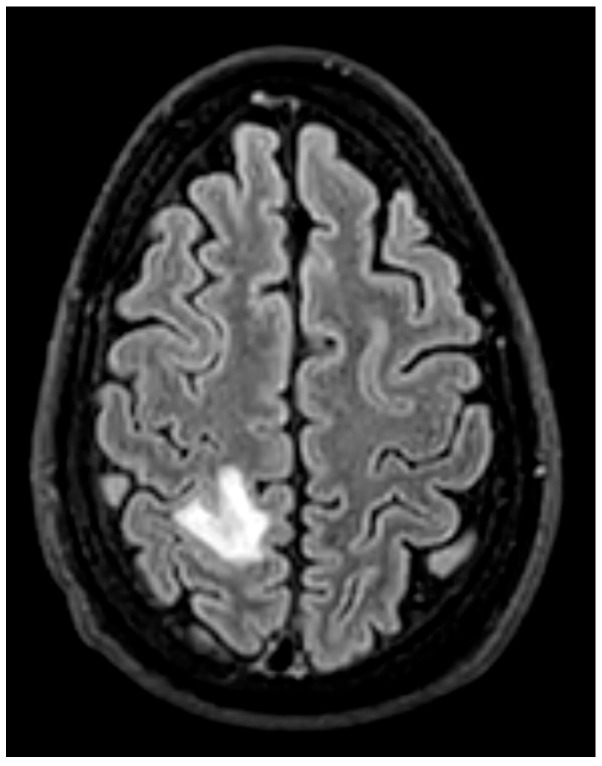
This patient had native aortic valve endocarditis. No neurological symptoms reported; brain magnetic resonance imaging was performed before surgery and showed an embolic lesion in the occipital cortex. This is an original, de-identified clinical image from our institution. Patient consent for publication was obtained.

- **Brain MRI** is especially valuable for identifying clinically occult cerebral emboli, which can influence management decisions and prognosis [24,36] (Figure 15). These occur in 20–40% of IE cases, and a significant portion are clinically silent. Brain MRI with diffusion-weighted imaging (DWI) is the most sensitive modality for detecting acute cerebral infarcts, microabscesses, mycotic aneurysms, and hemorrhages [35,38]. Identifying these findings, even in asymptomatic patients, is critical as it can significantly influence surgical timing and risk stratification. The 2023 ESC guidelines recommend considering brain MRI before cardiac surgery in patients with IE, especially in those with neurological symptoms or high-risk clinical features [13]. For example, the presence of a large cerebral infarct, hemorrhagic lesions, or mycotic aneurysms may prompt delay of valve surgery to reduce the risk of perioperative neurological complications. Conversely, detection of small, non-hemorrhagic embolic lesions may support earlier surgery to prevent further embolization [30]. Prospective data demonstrate that routine cerebral MRI led to changes in surgical plans in up to 14% of cases, including both delays and accelerations of surgery based on neurological risk [39].
- **Whole-Body MRI**: While less established than PET/CT, whole-body MRI can be used to detect embolic phenomena in other organs, such as the spleen and kidneys, particularly in patients for whom radiation exposure is a major concern [40].

### 6.2. The Complementary Role in Cardiac Assessment

Cardiac Magnetic Resonance (CMR) does not have a primary role in the initial diagnosis of IE due to its lower spatial resolution for small vegetations compared to transesophageal echocardiography (TEE) and longer acquisition times [38,41]. However, it is a valuable problem-solving tool in specific scenarios:

- Assessment of Perivalvular Complications: Late gadolinium enhancement (LGE) CMR can help characterize perivalvular tissue. In cases where echocardiography and CT are inconclusive, CMR can identify perivalvular abscesses or pseudoaneurysms by demonstrating a core of hyperintense signal on T2-weighted images (edema) with peripheral LGE (inflammatory capsule) [38,41].
- Myocardial Tissue Characterization: CMR is uniquely capable of identifying associated IE-related myocarditis or myocardial microabscesses through T2-weighted imaging (edema) and LGE, findings that are typically missed by other modalities [41,42].
- Comprehensive Functional Assessment: CMR provides the reference standard for quantifying biventricular volumes, ejection fraction, and valvular regurgitation. This is particularly useful for serial monitoring of ventricular function during and after treatment, especially in cases of associated valve dysfunction or heart failure.

### 6.3. Contrasting the Role of MRI/CMR with CT and PET/CT

It is crucial to understand the distinct niches of each advanced modality:

- vs. Cardiac CT: Cardiac CT is superior for detailed anatomical delineation of paravalvular structures, calcifications, and pseudoaneurysms due to its higher spatial resolution. It is the preferred modality for pre-surgical coronary angiography. CMR, however, provides superior soft-tissue characterization (differentiating edema, necrosis, and thrombus) and functional data without radiation [17,38].
- vs. FDG-PET/CT: PET/CT excels at detecting metabolic activity, making it superior for diagnosing active infection around prosthetic material and identifying occult septic emboli throughout the body. MRI does not assess metabolism but provides exquisite anatomical detail of embolic lesions (e.g., defining the exact size and hemorrhagic component of a splenic infarct) and can detect complications like cerebral microabscesses with higher sensitivity than CT [17,18].

In summary, MRI/CMR is not a first-line tool for the diagnosis of IE but is an indispensable adjunct for comprehensive patient management. Its primary strength lies in diagnosing silent cerebral and other embolic complications, while CMR serves as a powerful problem-solving tool for ambiguous perivalvular findings and for assessing myocardial tissue involvement and ventricular function.

## 7. Nuclear Imaging

**Figure 16:**
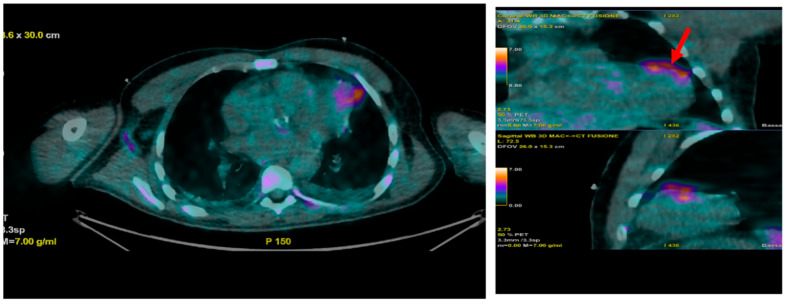
This patient was referred to the cath lab for chest pain with ST-segment elevation on ECG. Coronary angiography excluded obstructive coronary artery disease. Echocardiogram showed aortic valve vegetation, and suspected embolic phenomena led us to perform a PET/CT scan. A myocardial abscess was detected (arrow), probably secondary to septic embolization and causative of the ST-segment elevation. This is an original, de-identified clinical image from our institution. Patient consent for publication was obtained.

**Figure 17:**
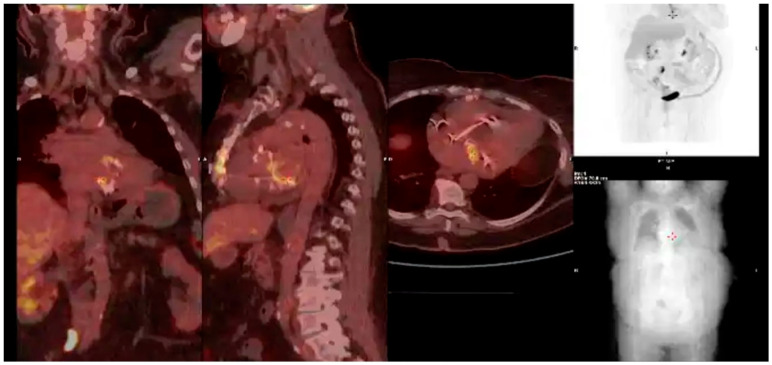
PET/CT findings in a patient with infective endocarditis involving a mechanical aortic prosthesis. This image shows tracer uptake at the level of the aortic prosthesis. This is an original, de-identified clinical image from our institution. Patient consent for publication was obtained.

**Figure 18:**
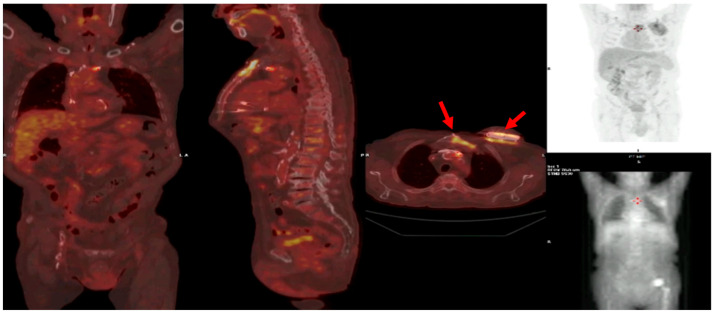
Patient with CIED endocarditis showing intense radiotracer uptake at the device pocket and catheter (red arrows) on this PET/CT scan. This is an original, de-identified clinical image from our institution. Patient consent for publication was obtained.

According to the 2023 European Society of Cardiology (ESC) guidelines, nuclear imaging with fluorodeoxyglucose positron emission tomography/computed tomography (FDG-PET/CT) and white blood cell (WBC) scintigraphy is indicated in the following scenarios for infective endocarditis (IE):

- Prosthetic valve endocarditis (PVE): FDG-PET/CT and WBC scintigraphy are recommended when echocardiography is inconclusive or negative, but clinical suspicion remains high. These modalities are especially valuable for detecting perivalvular infection, invasive complications, and extracardiac septic emboli. Abnormal focal uptake around the prosthesis is considered a significant criterion for PVE diagnosis, and can reclassify cases from “possible” to “definite” IE within the modified Duke criteria framework [13,43,44,45]
- Cardiac device infections: Nuclear imaging is indicated for suspected infection of cardiac implantable electronic devices (CIEDs) when conventional imaging is non-diagnostic. FDG-PET/CT can identify device pocket infection, lead infection, and associated extracardiac complications, and is integrated into the diagnostic algorithm for device-related IE [46]
- Reclassification from possible to definite IE: The ESC guidelines incorporate abnormal FDG-PET/CT or WBC scintigraphy findings as a significant criterion for IE diagnosis in patients with prosthetic valves or devices. This allows for reclassification of cases initially deemed “possible” IE to “definite” IE when nuclear imaging demonstrates focal uptake consistent with infection, particularly in the setting of non-diagnostic echocardiography or ambiguous clinical findings (Figure 16, Figure 17 and Figure 18) [43,44,46].

The ESC emphasizes that nuclear imaging should **be performed early**, ideally before prolonged antibiotic therapy, as sensitivity decreases with ongoing treatment. These recommendations are consistent with the diagnostic algorithms and criteria outlined by the European Society of Cardiology [13].

### Advanced Imaging in Cardiac Implantable Devices 

For cardiac implantable electronic devices (CIEDs), the ESC guidelines advise initial evaluation with echocardiography (preferably TEE), but recognize its limitations in distinguishing infectious from non-infectious lead masses.

The ESC guidelines recommend the use of additional imaging modalities, such as FDG PET/CT and white blood cell single-photon emission computed tomography/computed tomography (WBC SPECT/CT).

FDG PET/CT is especially valuable for identifying pocket infections, lead infections, and extracardiac complications such as septic emboli. It is now incorporated as a significant criterion in the ESC diagnostic algorithm for device-related endocarditis [47].

FDG PET/CT demonstrates high specificity (up to 98%) but variable sensitivity—ranging from as low as 38.5% for lead infection to as high as 80% for pocket infection—in evaluating suspected CIED infection. Its diagnostic accuracy is highest for generator pocket infections and lower for lead infections, where a negative scan does not rule out infection if clinical suspicion remains high. The positive predictive value is substantial, but the negative predictive value is limited, particularly for lead-related endocarditis or in patients who have previously received antibiotics, which can reduce sensitivity [48,49].

WBC SPECT/CT offers higher specificity (up to 100%) but lower sensitivity (around 60%) compared to FDG PET/CT. It is beneficial for confirming infection when FDG PET/CT is equivocal, but its clinical utility is limited by availability, cost, and less robust supporting evidence. A negative WBC SPECT/CT scan can reliably exclude CIED infection [50].

Cardiac CT may also be considered for anatomical assessment, particularly for detecting abscesses or complications not well visualized by echocardiography. However, its role in lead infection is limited due to metal artifact [51].

For detailed information on patient preparation and pitfalls in interpretation, particularly regarding post-surgical inflammation, see Section 10.1.

A diagnosis of definite CIED infection is made if there is a combination of clinical, microbiological, and pathological findings, along with at least one logical, primary pathological finding (from echocardiography or advanced nuclear imaging) according to the ESC guidelines.

## 8. Multimodality Imaging and Clinical Synergy: From Diagnosis to Management

### 8.1. Constructing the Diagnostic Puzzle

**Figure 19:**
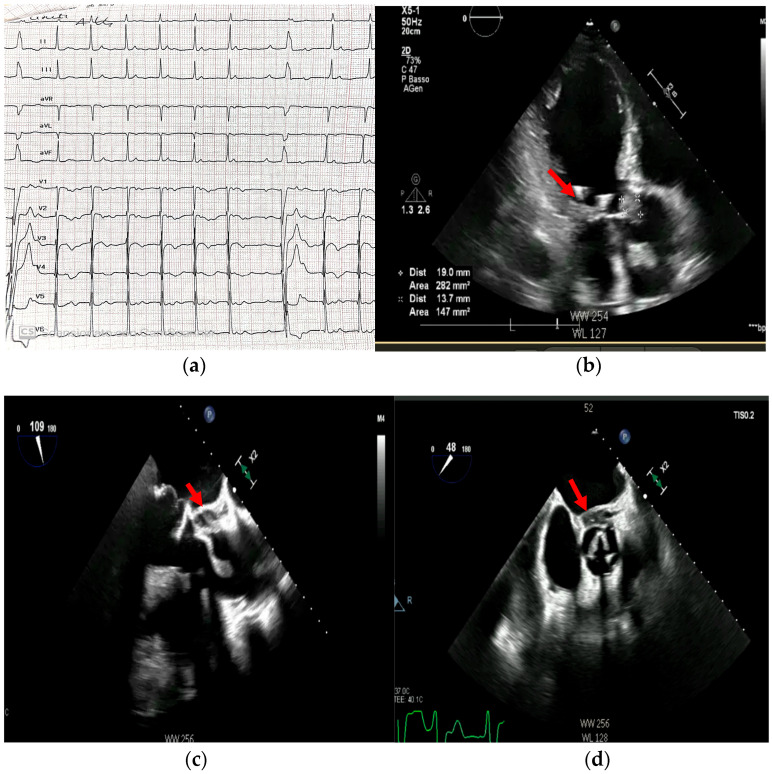
(**a**–**d**): ECG and echocardiogram findings of a patient with infective endocarditis of his bioprosthetic aortic valve (the patient also had a bioprosthetic mitral valve). The ECG shows atrioventricular dissociation suggestive of new-onset third-degree AV block (**a**), while the transthoracic echocardiogram shows a large aortic vegetation (**b**, arrow). The prompt execution of a transesophageal echocardiogram confirmed the presence of the vegetation (**c**, arrow) and revealed an anterior perivalvular abscess (**d**, arrow). Blood cultures were positive for *S. aureus* infection. The patient later underwent aortic valve replacement with discrete outcomes. This is an original, de-identified clinical image from our institution. Patient consent for publication was obtained.

Combining diagnostic modalities provides incremental value in diagnosing and managing infective endocarditis (IE), especially in complex cases (Figure 19).

Transthoracic echocardiography remains the first-line imaging modality, with TEE preferred for its higher sensitivity in detecting vegetations and intracardiac complications. However, limitations exist, particularly in prosthetic valve endocarditis and device-related infections, where artifacts and poor visualization can obscure findings. Cardiac CT offers superior anatomical resolution for paravalvular complications, while PET/CT can detect metabolic activity associated with infection, aiding in cases with negative or inconclusive echocardiography [4,12,52].

The 2023 ESC criteria incorporate these advanced primary diagnostic criteria, resulting in improved sensitivity (up to 69%) compared to the 2015 version, though specificity remains high (98%). This approach reduces misclassification of possible IE and facilitates earlier, more accurate diagnosis, which is critical for guiding management decisions such as the need for surgery or prolonged antimicrobial therapy.

For evaluation of extracardiac infectious foci, such as septic emboli to the brain, spleen, or other organs, cardiac computed tomography (CT) and fluorodeoxyglucose positron emission tomography/computed tomography (FDG-PET/CT) may be considered. FDG-PET/CT can identify distant embolic lesions in up to 35% of patients and is particularly valuable in prosthetic valve and device-related endocarditis, where it can reclassify possible cases to definite in up to 40% of patients and guide management changes, especially when echocardiography is inconclusive [20,43,51].

In PVE, positive FDG-PET/CT findings are associated with a higher risk of major cardiac events, including death, recurrence, heart failure, and new embolic events (hazard ratio 2.7 for the composite endpoint; HR 7.5 for new embolic events) [53].

Cardiac CT offers sensitivity and specificity for periannular complications (abscess, pseudoaneurysm, fistula) comparable to TEE (sensitivity 80%, specificity 82%), and is less affected by prosthetic material artifacts. The ACC and the Society of Cardiovascular Computed Tomography recommend cardiac CT as a first-line modality for delineating perivalvular extension, mycotic aneurysms, and for preoperative planning in PVE, and as a class IIa recommendation for suspected paravalvular abscess when echocardiography is inadequate [26,37].

This multimodality strategy enables earlier and more accurate identification of patients requiring surgery, modification of antibiotic regimens, and detection of embolic events [17,43,44].

### 8.2. When Clinical Findings Reshape the Pathway: The Case of Systemic Embolism

The true power of multimodality imaging is realized when its findings are integrated with the patient’s clinical evolution. Systemic embolization, a common complication occurring in 20–50% of cases, represents a pivotal clinical event that actively shifts the management pathway, demanding an immediate and strategic imaging response.

The detection of clinical signs of peripheral embolism is not merely a diagnostic criterion but a critical trigger that alters management in two key domains:

#### 8.2.1. Triggering an Expeditious and Expanded Diagnostic Workup

The emergence of a clinical sign suggestive of embolism—such as a focal neurological deficit, splenic pain, hematuria suggestive of renal infarction, or acute limb ischemia—should immediately escalate the diagnostic process.

- Confirmation of IE: In a patient with suspected IE but an inconclusive initial echocardiography, a new embolic event significantly raises the pre-test probability. This should prompt an urgent repeat transesophageal echocardiography (TEE) and strongly warrant the use of advanced imaging to secure the diagnosis
- Search for Silent Emboli: A single clinically apparent embolus is often the “tip of the iceberg.” The discovery of one embolic event mandates a systematic search for other, silent emboli, particularly cerebral, which can drastically alter surgical risk and timing. As such, whole-body CT angiography or FDG-PET/CT is recommended to define the full embolic burden [13,39]. Brain MRI is the gold standard for detecting silent cerebral emboli and should be strongly considered before surgery in high-risk patients, even in the absence of neurological symptoms [22].

#### 8.2.2. Directly Influencing Surgical Indications and Timing

The 2023 ESC guidelines formally recognize recurrent embolic events as a Class I indication for surgery (Level of Evidence B) [13].

- Recurrent Emboli: The occurrence of a second, clinically recognizable embolic event during appropriate antibiotic therapy is a strong indication for urgent surgery to remove the embolic source, irrespective of vegetation size or antibiotic course duration.
- Large Vegetation Size: While controversial, a persistent large vegetation (>10 mm) following a single embolic event is a Class IIa recommendation for surgery, reflecting the desire to pre-empt a potentially catastrophic recurrence [13].
- Impact of Silent Emboli: The discovery of multiple silent emboli, particularly cerebral, creates a complex risk-benefit calculus. While not a direct surgical indication, it necessitates a nuanced, multidisciplinary decision regarding the timing of surgery to balance the risk of recurrent embolism against the risk of hemorrhagic transformation of cerebral infarcts.

**Figure 20:**
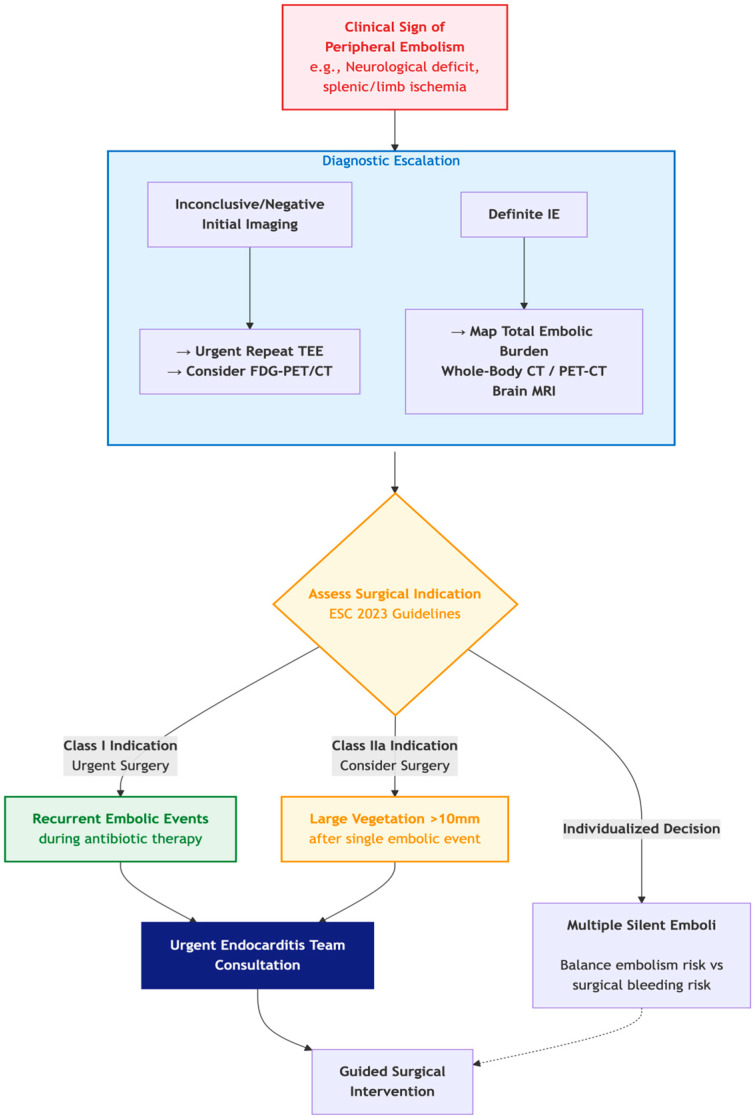
This figure summarizes the impact of peripheral emboli on management and re-definition of clinical urgency. This figure was originally created for this publication.

In conclusion, clinical signs of peripheral embolism are not static diagnostic checkboxes but dynamic clinical triggers. Therefore, the detection of peripheral embolism should activate a pre-defined protocol of escalated imaging and trigger an urgent Endocarditis Team consultation, perfectly illustrating the synergy between clinical vigilance, multimodality imaging, and multidisciplinary decision-making that defines modern IE care. The management shift triggered by embolism is summarized in Figure 20.

## 9. Multidisciplinary Approach

The endocarditis team is central to optimizing outcomes in infective endocarditis (IE), as emphasized by the European Society of Cardiology (ESC) 2023 guidelines. The ESC recommends a multidisciplinary approach involving cardiologists, cardiac surgeons, infectious disease specialists, microbiologists, and crucially, imaging specialists, to address the complex diagnostic and therapeutic challenges of IE [13,54].

This team-based model facilitates rapid, coordinated decision-making, especially in cases with diagnostic uncertainty, severe complications, or when surgical intervention is considered.

Imaging specialists play a pivotal role within the endocarditis team. Echocardiography remains the first-line modality for diagnosis and management. Still, advanced imaging—such as cardiac CT, PET/CT, and nuclear medicine techniques—is increasingly integrated, particularly for prosthetic valve endocarditis, device-related infections, and assessment of extracardiac complications [54,55,56].

The expertise of imaging specialists ensures appropriate selection, acquisition, and interpretation of multimodality imaging, which is essential for accurate diagnosis, detection of complications (e.g., abscess, embolic events), and surgical planning.

Observational studies and registry data suggest that the multidisciplinary endocarditis team approach improves time to intervention, reduces mortality, and enhances overall patient management, although randomized trial data are lacking [54,57].

The ESC 2023 guidelines specifically advocate for structured collaboration and regular multidisciplinary meetings to review cases, guide therapy, and coordinate follow-up, underscoring the indispensable role of imaging experts in this process.

## 10. Limitations, Evidence Gaps, and Future Research

While multimodality imaging is central to modern IE diagnosis, it is crucial to acknowledge that many recommendations, particularly those guiding the timing of surgery or the formation of endocarditis teams, are based on large observational cohorts, registry data, and expert consensus. The absence of randomized controlled trials comparing imaging-driven management strategies with conventional care remains a significant evidence gap. Consequently, the proposed algorithms, while reflecting best practices, are designed to integrate clinical judgment rather than replace it.

### 10.1. Pitfalls and Limitations of Advanced Imaging

- Echocardiography: As the cornerstone of diagnosis, echocardiography’s limitations in the setting of prosthetic material and operator dependence are well-known. The presence of sewing ring and stent frame shadowing can obscure critical findings, and Doppler flow malalignment may lead to underestimation of prosthetic valve gradients and regurgitation severity [37,58].
- FDG-PET/CT: Sterile post-surgical inflammation can cause increased FDG uptake, leading to false positives and reduced specificity within the first 1–3 months after valve implantation or cardiac surgery. Therefore, caution is advised when interpreting FDG-PET/CT results during this period [18,59]. Moreover, strict patient preparation is required in order to suppress physiological myocardial glucose uptake and improve diagnostic accuracy. A minimum 6 h fast and a low-carbohydrate, high-fat diet prior to imaging is needed, as this reduces background myocardial FDG uptake and enhances visualization of infectious foci. This stringent preparation can be challenging to achieve in acutely ill or diabetic patients, potentially limiting its real-world applicability.
- Cardiac CT: While excellent for anatomical definition, cardiac CT has limited sensitivity for small (<10 mm) mobile vegetations and cannot assess hemodynamic status. Its use is also limited by radiation exposure and the need for a stable sinus rhythm for accurate electrocardiographic gating, as arrhythmias can degrade image resolution and diagnostic yield [37,60,61]. Additionally, the use of iodinated contrast media carries a risk of nephrotoxicity, a relevant concern in a patient population often affected by chronic kidney disease.

### 10.2. Ongoing Controversies

- Standardized Protocols: Future research must focus on standardizing imaging protocols (e.g., patient preparation for FDG-PET/CT, standardized scoring systems, and Standardized Uptake Value measurements, describing pattern and distribution of uptake…) and reporting criteria to improve consistency and comparability across studies. Crucially, large, prospective, multicenter studies are needed to validate whether these advanced imaging strategies, when guided by a multidisciplinary team, translate into a measurable improvement in hard outcomes such as mortality, recurrent embolization, and need for reoperation.

### 10.3. Emerging Technologies

- Artificial intelligence (AI) and machine learning (ML) models have demonstrated promise in improving diagnostic accuracy for prosthetic valve endocarditis, with automated vegetation quantification enhancing risk stratification. Machine learning algorithms can predict postsurgical mortality and identify high-risk patients using imaging and biomarker data. Recent literature highlights that AI/ML can outperform traditional diagnostic methods in certain contexts, especially in complex cases such as prosthetic valve or device-related IE [62]. Current consensus supports the use of AI-enhanced imaging as a supplement to, not a replacement for, expert clinical judgment and multidisciplinary team-based decision-making in IE [17,21,55]. These technologies offer potential for real-time decision support and personalized management, but require rigorous external validation and assessment of clinical workflow integration before routine adoption [62].
- Novel radiotracers, such as 99mTc-HMPAO-SPECT/CT (Technetium-99m-Hexamethylpropyleneamine Oxime) and experimental PET agents, have demonstrated improved specificity for infection over inflammation in small cohorts, but their clinical utility and cost-effectiveness remain unproven in large, multicenter studies [63,64]. 99mTc-HMPAO-SPECT/CT provides additional specificity by directly imaging leukocyte accumulation at sites of active infection [51,64]. Emerging PET tracers target bacterial components or specific leukocyte subsets [65]. Hybrid molecular imaging, such as PET/MRI, is also being explored for simultaneous molecular and anatomical assessment, with potential advantages in soft tissue characterization and reduced radiation exposure [18].

### 10.4. Key Unanswered Questions Include

- What is the optimal timing and modality for follow-up imaging after initiation of treatment or device extraction?
- How can healthcare systems address the significant access disparities and cost barriers associated with advanced imaging modalities like FDG-PET/CT?
- And ultimately, do these advanced imaging strategies, when guided by a multidisciplinary team, translate into a measurable improvement in hard outcomes such as mortality and recurrent embolization? Most of the current data derives from observational studies, rather than randomized trials.

## 11. Summary and Recommendations

Multimodality imaging is central to the diagnosis and management of infective endocarditis (IE), with echocardiography as the first-line modality. Transthoracic echocardiography (TTE) is recommended for initial assessment. Still, transesophageal echocardiography (TEE) should be performed in many cases of suspected IE due to its superior sensitivity, especially for prosthetic valves, intracardiac devices, and paravalvular complications [1,2,3]. If initial echocardiography is negative but clinical suspicion remains high, repeat TEE is advised, especially if clinical hints of perivalvular involvement are present [1].

The European Society of Cardiology (ESC) 2023 guidelines emphasize integrating advanced imaging modalities when echocardiography is inconclusive or in complex cases. Cardiac computed tomography (CT) is particularly valuable for detecting paravalvular extension (abscess, pseudoaneurysm, fistula), prosthetic valve dehiscence, and for preoperative planning [4,5]. Nuclear imaging, such as 18F-FDG PET/CT and white blood cell scintigraphy, is recommended for prosthetic valve endocarditis and cardiac device infection, as it increases diagnostic sensitivity and can help reclassify possible IE to definite IE [2,5,6].

### 11.1. Practical Recommendations for Clinical Practice

- Always start with TTE, followed by TEE in suspected cases and specific populations (e.g, prosthetic heart valves).
- Use cardiac CT for suspected paravalvular complications or when TEE is non-diagnostic.
- Employ 18F-FDG PET/CT or WBC scintigraphy for prosthetic valve or device-related IE, and when blood cultures are negative but suspicion remains [2,5,6].
- Multidisciplinary “endocarditis teams” should coordinate imaging and management decisions [3,7]

**Figure 21:**
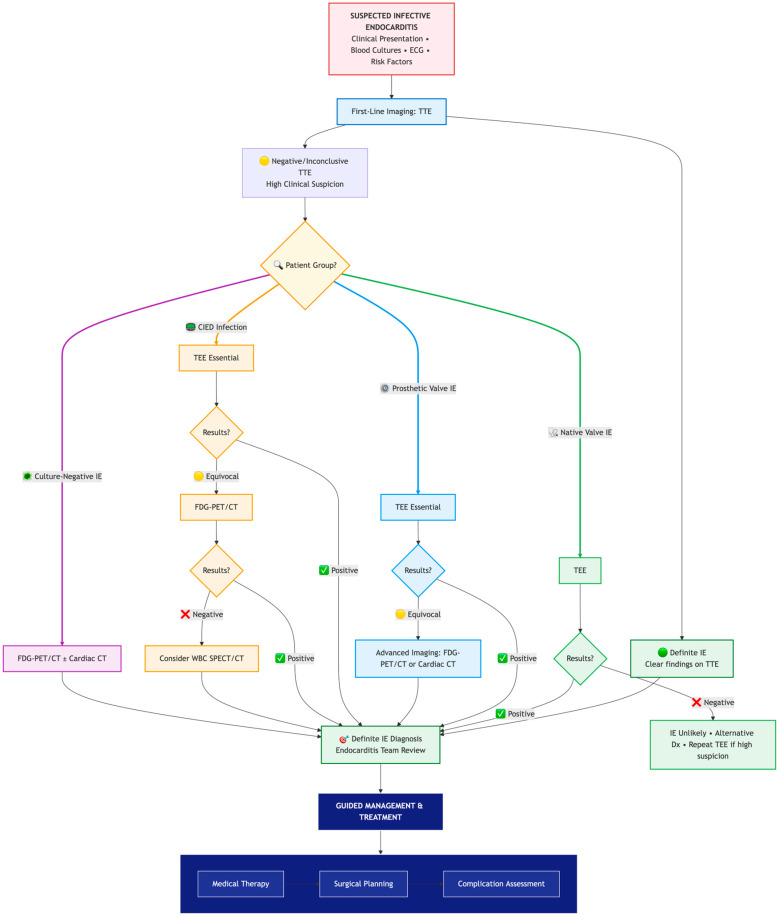
A stepwise, multimodality imaging approach to the diagnosis and management of infective endocarditis, integrating clinical presentation and the 2023 ESC guidelines. Abbreviations are as in text.

The ESC 2023 guidelines advocate a tailored, stepwise approach, integrating multimodality imaging to optimize diagnostic accuracy and guide therapy (Figure 21) [1,2,4,5,6]. For a quick-reference summary of the comparative strengths, limitations, and ideal use cases of each imaging modality, see Table 1.

### 11.2. Key Points

- **Diagnostic Hierarchy is Critical:** While transthoracic echocardiography (TTE) is the essential first-line test, transesophageal echocardiography (TEE) is often mandatory, and its limitations in the setting of prosthetic material must be actively recognized.
- **Scenario-Based Imaging Selection is Paramount:** The diagnostic pathway must be tailored, with early integration of advanced modalities like FDG-PET/CT and Cardiac CT for prosthetic valve and cardiac device-related infections, where they significantly increase diagnostic certainty.
- **Advanced Modalities Offer Complementary Data:** Cardiac CT excels at defining paravalvular anatomy for surgery, FDG-PET/CT detects occult metabolic activity and emboli, and MRI is indispensable for diagnosing silent cerebral complications.
- **Clinical Vigilance Drives Management Shifts:** The occurrence of systemic embolism is not just a diagnostic clue but a pivotal event that should trigger an escalated imaging protocol and urgent surgical evaluation.
- **A Multidisciplinary Endocarditis Team is Non-Negotiable:** Optimal patient outcomes depend on the collaborative interpretation of multimodality imaging findings within a dedicated team framework to guide complex therapeutic decisions.
- **Acknowledge the Evidence Landscape:** Practitioners must be aware of modality-specific pitfalls (e.g., PET/CT’s post-surgical inflammation, MRI’s limited spatial resolution) and that many recommendations are grounded in expert consensus, requiring integration with clinical judgment.

## Figures and Tables

**Table 1:** Comparison of Imaging Modalities in Infective Endocarditis.

Modality	Primary Strengths	Key Limitations	Ideal Use Case
**Transthoracic Echocardiography (TTE)**	**First-line,** rapid, & non-invasive Excellent for valvular function & hemodynamics High specificity for vegetations in NVE High negative predictive value in low-risk NVE	Limited sensitivity in PVE, CIED, obese patients Acoustic shadowing from prosthetic material Operator-dependent Poor for paravalvular anatomy detail	**Initial evaluation** of all suspected IE. **Ruling out IE** in low-probability native valve cases.
**Transesophageal Echocardiography (TEE)**	High sensitivity & specificity for vegetations, abscesses, perforations **Superior to TTE for PVE and CIED leads** Excellent for assessing valve dysfunction mechanisms Intraoperative guidance	Semi-invasive Sedation risk Still limited by significant prosthetic shadowing Cannot reliably distinguish vegetation from sterile thrombus on leads	**Definitive diagnosis when TTE is inconclusive**, but clinical suspicion is high. Evaluating **complications and guiding surgery.**
**Cardiac Computed Tomography (CT)**	Superior spatial resolution for **paravalvular anatomy** Excellent for abscess, pseudoaneurysm, fistula, dehiscence Less affected by prosthetic artifacts Pre-surgical planning (including coronary anatomy)	Poor for small, mobile vegetations Radiation exposure Requires stable rhythm for gating; contrast nephrotoxicity risk No hemodynamic assessment	Assessing **paravalvular complications** when TEE is inconclusive. **Pre-surgical planning** in complex PVE.
FDG-positron emission tomography/computed tomography **(FDG-PET/CT)**	Detects **metabolic activity** of infection **High specificity for PVE and CIED infection** (outside postoperative window) Whole-body screen for septic emboli & occult infection Can reclassify “possible” to “definite” IE	Low specificity in first 1–3 months post-op (sterile inflammation) Requires stringent patient preparation (fasting, diet) Reduced sensitivity with prolonged antibiotics Limited spatial resolution for small vegetations	**Prosthetic valve & CIED infection** with inconclusive echo. **Culture-negative IE** and search for metastatic infection.
**White Blood Cell SPECT/CT (WBC Scan)**	Very high specificity for infection (vs. inflammation) Useful when FDG-PET/CT is equivocal or contraindicated A **negative study can reliably exclude infection**	Lower sensitivity than FDG-PET/CT Logistically complex (requires leukocyte labeling) Limited availability; 24–48 h delay for results Poor spatial resolution	Confirming infection in complex PVE, especially **early post-op.** **Problem-solving tool after equivocal FDG-PET/CT.**
**Cardiac Magnetic Resonance (CMR)**	Reference standard for ventricular function & tissue characterization Can detect IE-related myocarditis/microabscesses No ionizing radiation	Not for primary IE diagnosis (low sensitivity for small vegetations) Long acquisition times; limited availability for acute cases May be contraindicated with CIEDs	**Quantifying ventricular function and impact of valvular lesions.** Detecting associated myocarditis.
**Brain/Whole-Body MRI**	Most sensitive modality for detecting **silent cerebral emboli**, microabscesses, mycotic aneurysms Critical for **pre-surgical risk stratification**	Does not assess cardiac structures for IE diagnosis May delay surgery if concerning findings are detected on brain imaging.	

Comparative strengths, limitations, and primary clinical applications of imaging modalities in the diagnosis and management of infective endocarditis (IE). The modalities are complementary, and their use should be tailored to the clinical scenario (NVE, PVE, or CIED-related IE). FDG-PET/CT: Fluorodeoxyglucose Positron-Emission Tomography/Computed Tomography; WBC SPECT/CT: White Blood Cell Single-Photon Emission Computed Tomography/Computed Tomography; CMR: Cardiac Magnetic Resonance; MRI: Magnetic Resonance Imaging; NVE: Native Valve Endocarditis; PVE: Prosthetic Valve Endocarditis; CIED: Cardiac Implantable Electronic Device.

## Data Availability

The data presented in this study are available on request from the corresponding author.

## Supplementary Materials

The following supporting information can be downloaded at: https://www.mdpi.com/article/10.3390/medicina61122241/s1, Table S1: Detailed search strategy for PubMed/MEDLINE.

## Abbreviations

IE	Infective Endocarditis
CIED	Cardiac implantable Electronic Devices
ESC	European society of cardiology
PET/CT	Positron Emission Tomography/Computerized Scan
AHA	American Heart Association
ACC	American College of Cardiology
TEE	Transesophageal Echocardiography
TTE	Transthoracic Echocardiography
ISCVID	International Society for CardioVascular Infectious Diseases
SPECT	Single Photon Emission Computed Tomography
AV	Atrioventricular
PVE	Prosthetic Valve Endocarditis
NVE	Native Valve Endocarditis
WBC	White Blood Cells
AI	Artificial Intelligence
ML	Machine Learning
99mTc	Technetium-99m
HMPAO	Hexamethylpropyleneamine Oxime)
MAIVF	Mitral-Aortic Intervalvular Fibrosa
LCA	Left Coronary Artery
BVP	Biological Valve Prosthesis
LVOT	Left Ventricular Outflow Tract
LA	Left Atrium
DWI	Diffusion-Weighted Imaging
MALDI	Matrix-Assisted Laser Desorption/Ionization
TOF	Time-of-Flight
NBTE	Nonbacterial thrombotic endocarditis
BCNE	Blood Culture Negative Endocarditis
