# Supplementary material for: Multimodality Imaging in Infective Endocarditis: A Clinical Approach to Diagnosis
Source: Medicina (Kaunas). 2025 Dec 18;61(12):2241. doi: 10.3390/medicina61122241 (PMC12735077; doi:10.3390/medicina61122241)
Supplement: Supplementary file 1 [file medicina-61-02241-s001.zip › medicina-4018173-supplementary.pdf]

Supplementary Table 1: Detailed search strategy for PubMed/MEDLINE, executed on [September 10, 2025].
Database: PubMed/MEDLINE
Date Range: January 1, 1980 - August 31, 2025
Filters Applied: English language, Human studies, Imaging studies

Line	Query
#1	"Endocarditis"[Mesh] OR "endocarditis"[tiab] OR "infective endocarditis"[tiab] OR "bacterial endocarditis"[tiab] OR "IE"[tiab]
#2	"Multimodal Imaging"[Mesh] OR "multimodality imaging"[tiab] OR "multimodal imaging"[tiab] OR "multi-modality imaging"[tiab] OR "hybrid imaging"[tiab]
#3	"Echocardiography"[Mesh] OR "echocardiography"[tiab] OR "echocardiogram"[tiab] OR "TTE"[tiab] OR "TEE"[tiab] OR "transesophageal echocardiography"[tiab] OR "transthoracic echocardiography"[tiab] OR "3D echocardiography"[tiab] OR "three-dimensional echocardiography"[tiab]
#4	"Tomography, X-Ray Computed"[Mesh] OR "computed tomography"[tiab] OR "cardiac CT"[tiab] OR "CT"[tiab] OR "computed tomographic"[tiab] OR "CCT"[tiab]
#5	"Positron-Emission Tomography"[Mesh] OR "positron emission tomography"[tiab] OR "PET"[tiab] OR "FDG-PET"[tiab] OR "PET/CT"[tiab] OR "PET-CT"[tiab] OR "18F-FDG"[tiab]
#6	"Magnetic Resonance Imaging"[Mesh] OR "magnetic resonance imaging"[tiab] OR "MRI"[tiab] OR "cardiac MRI"[tiab] OR "CMR"[tiab] OR "cardiac magnetic resonance"[tiab]
#7	"Radionuclide Imaging"[Mesh] OR "nuclear imaging"[tiab] OR "scintigraphy"[tiab] OR "SPECT"[tiab] OR "white blood cell scan"[tiab] OR "WBC scintigraphy"[tiab] OR "leukocyte scintigraphy"[tiab]
#8	#3 OR #4 OR #5 OR #6 OR #7
#9	#1 AND #2 AND #8
#10	#9 NOT ("pediatric"[tiab] OR "child"[tiab] OR "children"[tiab] OR "childhood"[tiab] OR "infant"[tiab] OR "adolescent"[tiab] OR "newborn"[tiab] OR "neonatal"[tiab])
#11	#10 Filters: English, Human, 1980/01/01:2025/08/31

Note: Similar search strategies were used for Embase and Scopus databases, adapting the controlled vocabulary (EMTREE for Embase) while maintaining the same conceptual structure.
